# miR-200a Modulates the Expression of the DNA Repair Protein OGG1 Playing a Role in Aging of Primary Human Keratinocytes

**DOI:** 10.1155/2018/9147326

**Published:** 2018-03-25

**Authors:** Lavinia Tinaburri, Mariarosaria D'Errico, Sara Sileno, Riccardo Maurelli, Paolo Degan, Alessandra Magenta, Elena Dellambra

**Affiliations:** ^1^Molecular and Cell Biology Laboratory, Istituto Dermopatico dell'Immacolata-IRCCS, Rome, Italy; ^2^Section of Mechanisms, Biomarkers and Models, Department of Environment and Health, Istituto Superiore di Sanità, Roma, Italy; ^3^Vascular Pathology Laboratory, Istituto Dermopatico dell'Immacolata-IRCCS, Rome, Italy; ^4^IDI Farmaceutici, Pomezia, Rome, Italy; ^5^U.O. Mutagenesi e Prevenzione Oncologica, Ospedale Policlinico San Martino, Genova, Italy

## Abstract

Oxidative DNA damage accumulation may induce cellular senescence. Notably, senescent cells accumulate in aged tissues and are present at the sites of age-related pathologies. Although the signaling of DNA strand breaks has been extensively studied, the role of oxidative base lesions has not fully investigated in primary human keratinocyte aging. In this study, we show that primary human keratinocytes from elderly donors are characterized by a significant accumulation of the oxidative base lesion 8-OH-dG, impairment of oxidative DNA repair, and increase of miR-200a levels. Notably, OGG1-2a, a critical enzyme for 8-OH-dG repair, is a direct target of miR-200a and its expression levels significantly decrease in aged keratinocytes. The 8-OH-dG accumulation displays a significant linear relationship with the aging biomarker p16 expression during keratinocyte senescence. Interestingly, we found that miR-200a overexpression down-modulates its putative target Bmi-1, a well-known p16 repressor, and up-regulates p16 itself. miR-200a overexpression also up-regulates the NLRP3 inflammasome and IL-1*β* expression. Of note, primary keratinocytes from elderly donors are characterized by NRPL3 activation and IL-1*β* secretion. These findings point to miR-200a as key player in primary human keratinocyte aging since it is able to reduce oxidative DNA repair activity and may induce several senescence features through p16 and IL-1*β* up-regulation.

## 1. Introduction

Skin protects the body from several environmental stressors. However, this protection can be compromised by skin aging that is characterized by accumulation of macromolecular damage and senescent cells, inflammation, and impaired tissue renewal and repair [1].

Cellular senescence is induced by several stimuli that lead to growth arrest and typical phenotypic alterations, including chromatin and secretome changes. Indeed, senescent cells acquire a proinflammatory status defined as senescence-associated secretory phenotype (SASP), characterized by the secretion of several growth factors, cytokines, and extracellular proteases that participate in tissue remodeling, modifying the cellular microenvironment [2]. Therefore, cellular senescence impedes the uncontrolled proliferation of damaged cells and promotes the tissue clearance and regeneration. However, in old organisms, the continuous and cumulative damages and the reduced clearance of senescent cells result in their accumulation with detrimental effects on tissue homeostasis [3–5]. This chronic proinflammatory status creates a tissue microenvironment that is permissive for tumor development and seems to be the major contributor to the increase of cancer incidence and progression in aged people. Thus, aging may be considered a major risk factor for skin malignant transformation [5].

Epidermis is continuously renewed during life, and keratinocyte stem cells contribute to normal tissue homeostasis and regeneration in response to injury or stress. To replace keratinocytes lost during tissue turnover, some stem cells daughters are committed to differentiate. They become transient amplifying (TA) cells that accomplish several cycles of division before withdrawing from the cell cycle to execute the terminal differentiation program [6]. This transition, from stem to TA cells and to terminally differentiated paraclones, is named clonal evolution. Epidermal aging occurs as result of both the genetic program and the cumulative oxidative stress induced by oxidative metabolism and environmental stresses (e.g., UV radiations) that may induce cellular senescence [7]. Oxidative stress originates from an imbalance between the generation of reactive oxygen species (ROS) and their scavenging by antioxidant defenses. Massive formation of ROS results in the damage of macromolecules (e.g., nucleic acids, proteins, and lipids) that is potentially deleterious to cells [1, 8]. Thus, these cellular components have to be protected against oxidation by several defense mechanisms, such as DNA repair systems [9]. Keratinocytes are provided of special mechanisms for ROS scavenging compared to fibroblasts and an efficient global genome repair that is preserved also in terminally differentiated cells [1, 10]. However, the increased generation of ROS and/or reduced activity of host defense mechanisms during aging may induce an accumulation of oxidative damage in nuclear and mitochondrial DNA contributing to age-related genomic instability. This DNA damage leads to persistent DNA damage response, which drives the cells to undergo senescence and acquire a SASP [3, 11]. Albeit signaling by DNA strand breaks convincingly mediates keratinocyte senescence [12], the role of oxidative base lesions is however not fully understood in this process.

In nuclear and mitochondrial DNA, 8-hydroxy-2′-deoxyguanosine (8-OH-dG) is one of the predominant forms of ROS-induced oxidative lesions. The 8-OH-dG accumulation has been associated with aging and age-related diseases, including cancer [13–22]. Indeed, the 8-OH-dG lesion is highly mutagenic as yields a GC-to-TA transversion upon its replication by a DNA polymerase [23]. Thus, 8-OH-dG has been widely used as a biomarker of DNA oxidative damage and carcinogenesis [24]. Oxidative base lesions are mainly repaired by the base excision repair (BER) pathway. The BER is a multistep process that engages various proteins and is characterized by the following major steps: (i) recognition and excision of an inappropriate base by a DNA glycosylase, (ii) incision at the resulting abasic site by an endonuclease, (iii) replacement of the excised nucleotide, (iv) processing of the terminal end(s), and (v) sealing of the nick by a DNA ligase [25]. The 8-OH-dG lesion is predominantly identified and released by the 8-oxoguanine DNA glycosylase/AP lyase (OGG1). In aged cells, the increase of 8-OH-dG levels may result from the supraphysiological levels of ROS and/or decrease in OGG1 activity [26].

A reverse correlation exists between OGG1 activity and oxidative stress [27]. OGG1 seems to possess critical redox-sensitive residues whose reduced state is important for its glycosylase activity [28]. Moreover, OGG1 activity may be increased by acetylation that results from the unbalanced action of the redox-sensitive histone acetyltransferases versus deacetylase enzymes [27]. Oxidative stress induces also the expression of several small noncoding RNA molecules (containing about 21–23 nucleotides) named microRNAs (miRNAs) [29] that are critical posttranscriptional regulators of specific target genes, such as those involved in cell senescence and inflammation [30]. Although posttranslational modulation of OGG1 activity following ROS increase has been extensively studied [27], only few data concerning OGG1 regulation by miRNA expression are available [31, 32].

The aim of this study was, therefore, to investigate the role of oxidative DNA damage repair system in age-related epidermal cell senescence and skin inflammation.

## 2. Materials and Methods

### 2.1. Cell and Culture Conditions

Primary keratinocyte cultures were established from biopsies of the skin from 20 healthy donors (10 young subjects 2–45 years and 10 old subjects 60–82 years). Keratinocytes were cultivated on a feeder layer of lethally irradiated 3T3-J2 cells (a gift from Prof. H. Green), as described [33]. Briefly, skin biopsies were minced and trypsinized (0.05% trypsin/0.01% EDTA) at 37°C for 3 hours. Cells were collected every 30 minutes, plated on lethally irradiated 3T3-J2 cells, and cultured in 5% CO_2_ and humidified atmosphere in keratinocyte growth medium: DMEM and Ham's F12 media (3 : 1 mixture) containing fetal calf serum (FCS, 10%), insulin (5 *μ*g/ml), adenine (0.18 mM), hydrocortisone (0.4 *μ*g/ml), cholera toxin (CT, 0.1 nM), triiodothyronine (2 nM), epidermal growth factor (EGF, 10 ng/ml), glutamine (4 mM), and penicillin-streptomycin (50 IU/ml).

For serial propagation, cells were passaged at the stage of subconfluence, until they reached senescence [34]. For colony forming efficiency (CFE) assay, cells (100–1000) from each biopsy and from each cell passage were plated onto 3T3 feeder layers and cultured as above. Colonies were fixed 14 days later, stained with rhodamine B, and scored under a dissecting microscope. Colony forming efficiency values are expressed as the ratio of the number colonies on the number of inoculated cells. Paraclone percentage was calculated as the ratio of the number of aborted colonies on the number of total colonies [34, 35].

The number of cell generations was calculated using the following formula: *x* = 3.322 log *N*/No, where *N* is the total number of cells obtained at each passage and No is the number of clonogenic cells. Clonogenic cells were calculated from CFE data, which were determined separately in parallel dishes at the time of cell passage [34, 35]. Life span percentage was calculated as the ratio of the number of cell doublings (at selected passage) on the total number of cell doublings.

The human embryonic kidney (HEK) 293 cells (ATCC) and primary human fibroblasts, established from skin biopsies from healthy young donors, were grown in Dulbecco's modified Eagle's medium (DMEM) supplemented with 10% fetal bovine serum (FBS; Euroclone).

### 2.2. Measurement of Intracellular ROS Levels

Intracellular ROS levels were determined using the oxidation-sensitive fluorescent probe 2′,7′-dichlorofluorescein diacetate (H2 DCFDA) and detected by flow cytometry [36]. The conversion of H2 DCFDA into a fluorescent molecule is proportional to the ROS concentration.

### 2.3. Measurement of 8-OH-dG

8-OH-dG was determined by HPLC with electrochemical detection (HPLC/EC) as described previously [37] following DNA extraction, RNase treatment, and enzymatic hydrolysis. The levels of 8-OH-dG were referred to the amount of deoxyguanosine (dG) detected by UV absorbance at 254 nm. The levels of 8-OH-dG were expressed as the number of 8-OH-dG adducts per 10^6^dG bases (8-OH-dG/10^6^dG).

### 2.4. In Vitro Repair Assays

In vitro DNA repair assays with cell extracts were performed as previously described [38]. Briefly, the extracts from young and old donors were tested using Rhodamine Green-labelled plasmid substrates containing 8-OH-dG. After incubation, plasmid DNA was submitted to SDS-PAGE to detect the fluorescence intensity of full-length DNAs and cleaved fragments by image analysis.

### 2.5. miRNA Target Prediction and Luciferase Assay

Computational prediction of OGG1 as target gene of miR-33a and miR-200a was done using published algorithms: TargetScan (http://www.targetscan.org; Envisioneering Medical Technologies) and miRanda (http://www.microrna.org/; Memorial Sloan Kettering Cancer Center).

The demonstration that OGG1-2a is a direct target of miR-200a was successively verified by luciferase assays. The 3′-UTR sequence of the OGG1 gene (NM_016821.2) inserted in a firefly/Renilla duo-luciferase reporter vector (pEZX-MT06) was obtained by GeneCopoeia. HEK 293 cells were seeded in 12-well plates and cotransfected with 0.1 *μ*g of OGG1 3′-UTR target sequence expression plasmid with 0.25 *μ*g of pre-miR-200a or miR-scramble. Cellular extracts were tested with dual-luciferase assay (Promega) according to the manufacturer's instructions, using a Victor3 1420 Multilabel Counter (PerkinElmer). Values were normalized according to Renilla luciferase activity.

### 2.6. miR-200a Overexpression

Transient expression of miR-200a or miR-scramble in HEK 293 cells and primary human fibroblasts was carried out. Cells were seeded onto 9.6 cm^2^ wells (60.000 cells/well) and transfected with 80% confluence. Transfection was performed for 6 h in medium containing Lipofectamine 2000 (Invitrogen Life Technologies, CA, USA) and DNA (in the range of 2.5–10.5 *μ*g). Transfection assays were performed in triplicate. Transfection medium was then removed, and cells were allowed to recover in fresh medium for 48 h. Cultures were harvested for further analyses. miR-200a overexpression was controlled by quantitative real-time PCR (qRT-PCR) (see methods below).

### 2.7. Quantitative RT-PCR

RNA was extracted from cells using the TRIzol (Invitrogen).

For mRNA quantification, total RNA was reverse transcribed using an oligo(dT) primer (Invitrogen). mRNA levels were analyzed by QuantiTect SYBR Green PCR kit (QIAGEN) using ABI PRISM 7000 (Applied Biosystems). mRNA levels were normalized using the GUSB and GAPDH genes as housekeeping genes. The following primer sets were used (see Table 1).

For the determination of the expression level of selected genes of DNA repair systems using the TaqMan Low Density Arrays method (Applied Biosystems).

For miRNA quantification, total RNA was reverse transcribed using TaqMan microRNA Reverse Transcription Kit (Life Technologies). Primers for miR-33a and miR-200a were obtained from Life Technologies. miRNA levels were analyzed by the TaqMan microRNA Assays (Life Technologies) using ABI PRISM 7000. miRNA expression was normalized using the U6snRNA as control.

Relative expressions were calculated using the comparative Ct method (2^−ΔΔCt^).

### 2.8. Western Blot Analysis

For immunoblots, cells were extracted on ice with lysis radioimmunoprecipitation assay (RIPA) buffer and equal amounts of samples (50 *μ*g) were electrophoresed on 12.5% SDS-polyacrylamide gels (PAGE). Western blot was performed as described in [34], using the following anti-caspase 1 (Santa Cruz Biotechnology), anti-Bmi-1 (Upstate), anti-p16 (Abcam), anti-GAPDH (Santa Cruz Biotechnology), and anti-*β*-tubulin (Abcam) antibodies.

### 2.9. IL-1*β* Secretion

For IL-1*β* secretion analysis, primary keratinocytes from young and old donors were cultured for 72 h in starvation medium. Primary human fibroblasts and HEK 293 cells were cultured for 48 h in starvation medium after transduction. Media were collected and submitted to 3 biotin-label-based antibody array for profiling of protein secretion (RayBio® C-Series Human Cytokine Antibody Array C6). Protein levels were evaluated by densitometric analysis using a GS-710 scanner and Quantity One software (Bio-Rad). Data analysis was performed as described in [39].

### 2.10. Statistical Analyses

Each quantitative variable was checked for normality distribution using D'Agostino and Pearson omnibus normality test. Since most of the variables had a nonnormal distribution, correlation analysis was carried out using a Spearman test. Linear or nonlinear regression analysis has been used to determine the best relationship between data sets. Statistical analysis was performed using GraphPad Prism 5.0.

In histograms, all data were expressed as mean ± standard error of the mean (SEM) and analyzed by Student's test. *p* < 0.05 was considered statistically significant.

## 3. Results

### 3.1. Oxidative Damage in Aged Primary Human Keratinocytes

The age-dependent accumulation of 8-OH-dG may result from a decline of antioxidant defense and DNA repair activities and/or an increase of intracellular ROS [8]. However, the accumulation of 8-OH-dG with aging is not a continuous event in most tissues. Indeed, no significant increase has been observed until the end of middle age. The steady-state level of 8-OH-dG abruptly increased in elderly donors [26, 40, 41]. Although 8-OH-dG accumulation has been found in many aged tissues, to the best of our knowledge, there are no data concerning the human epidermis.

**Figure 1:**
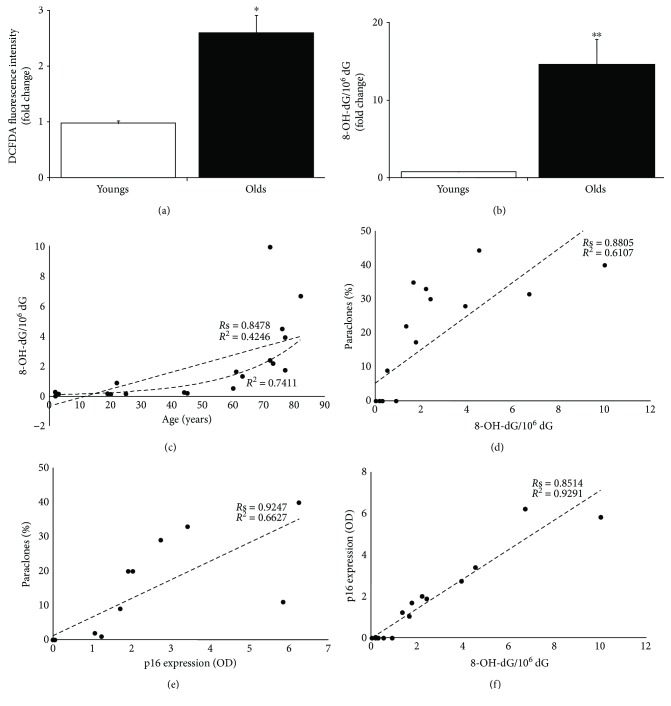
Oxidative damage in aged primary human keratinocytes. (a) Intracellular ROS steady-state levels were determined incubating keratinocytes from young and old donors (at 2nd passage) with 2′,7′-dichlorofluorescein diacetate (H2 DCFDA) and detected by flow cytometry. ROS level was given by fluorescence intensity per 10^3^ cells. Data were shown as fold change (*n* = 3, ^∗^ *p* < 0.05 by Student's test). (b) DNA was isolated from young and old donors (at 2nd passage), and the 8-OH-dG residue amount was determined by HPLC-ED. 2′-Deoxyguanosine (dG) was measured in the same run of corresponding 8-OH-dG, and the results were expressed as the number of 8-OH-dG residues/10^6^dG residues. Data were shown as fold change (*n* = 10, ^∗∗^ *p* < 0.01 by Student's test). (c–f) The best-fit line determined by linear regression was shown for each data series, with *R* ^2^ coefficient and Spearman correlation coefficient (*R*s). Two-tailed *p* value was reported in Result. Densitometric value of p16 expression was referred to Western blots shown in [35].

The levels of intracellular ROS in primary human keratinocytes were measured by using 2′,7′-dichlorofluorescein diacetate (DCFH-DA) as a probe and by monitoring its oxidation by flow cytometry. The steady-state ROS levels were 2.66-fold higher (*p* < 0.05) in primary keratinocytes from old donors as compared to young ones (Figure 1(a)). Thus, aged keratinocytes display alterations of the cellular redox balance in physiological conditions.

The levels of 8-OH-dG residues were measured in primary human keratinocytes obtained from 10 young donors (2–45 years) and 10 old donors (60–82 years) using high-performance liquid chromatograph/electrochemical detection (HPLC-EC) methodology. As shown in Figure 1(b), the steady-state level of 8-OH-dG was significantly higher in cultures from old donors. Keratinocytes from old donors displayed a 19.42-fold increase (*p* < 0.01) of 8-OH-dG lesions compared to cultures from young ones. Notably, the strongest 8-OH-dG increase was observed in elderly donors (>70 years) (Figure 1(c)). A high significant positive correlation was found between donor age and 8-OH-dG (Figure 1(c); *R*s = 0.8478, *p* < 0.0001) with a moderate linear relationship (Figure 1(c); *R* ^2^ = 0.4246, *p* = 0.0019). However, the exponential curve fits the data better than the linear curve (Figure 1(c); *R* ^2^ = 0.7411) demonstrating that the accumulation of 8-OH-dG with aging is not a linear event also in human skin.

A high significant positive correlation was found between 8-OH-dG and paraclone percentage (Figure 1(d); *R*s = 0.8805, *p* < 0.0001), which is an index of clonal evolution accomplishment and, in turn, stem cell depletion [34]. 8-OH-dG and paraclone percentage displayed a significant linear relationship (Figure 1(d); *R* ^2^ = 0.6107, *p* < 0.0001).

Keratinocyte clonal evolution is driven by progressive accumulation of the cell cycle inhibitor p16, also named p16^INK4a^ [42], that is considered one of the most robust aging biomarkers [43–46]. Data from progeroid and calorically restricted rodents and human tissues suggest that p16 may be a marker of biological rather than chronological aging [46, 47]. Senescence features of these keratinocyte cultures have been previously analyzed [35]. Specifically, paraclone percentage displayed a significant positive correlation with p16 increase at first in vitro passage (Figure 1(e); *R*s = 0.9247, *p* < 0.0001) with a linear relationship (Figure 1(e); *R* ^2^ = 0.6627, *p* < 0.0001).

Here, we found a strong significant positive correlation between 8-OH-dG levels and p16 expression (Figure 1(f); *R*s = 0.8514, *p* < 0.0001). Notably, 8-OH-dG levels and p16 expression displayed a strong significant linear relationship (Figure 1(f); *R* ^2^ = 0.9291, *p* < 0.0001).

Thus, the keratinocytes from elderly donors are characterized by a significant increase of 8-OH-dG levels. This increase displays a linear relationship mainly with the tissue senescence instead of chronological individual aging. Altogether, these data point to 8-OH-dG as a biomarker of human skin aging.

### 3.2. Oxidative Damage in Senescent Primary Human Keratinocytes

Accumulation of senescent cells is thought to contribute to biological aging of tissues. Cellular senescence refers to both premature and replicative senescence. However, both types of senescence display overlapping phenotypes and molecular effectors [48].

**Figure 2:**
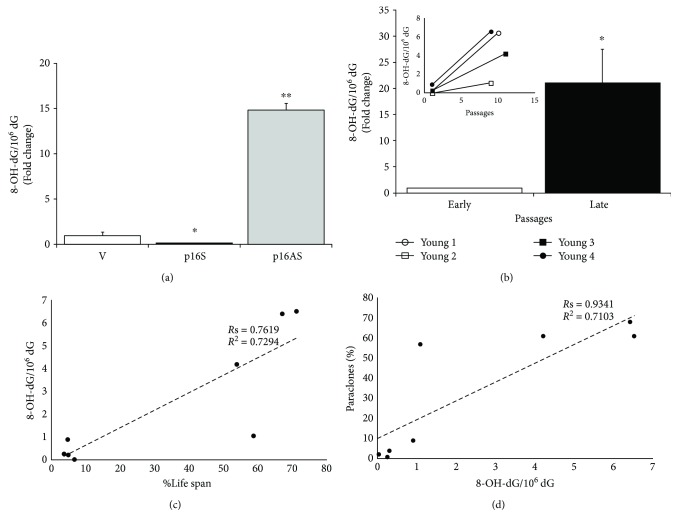
Oxidative damage in senescent primary human keratinocytes. (a) DNA was isolated from empty vector-, p16 sense-, and p16-antisense-transduced primary human keratinocyte (at 2nd passage) [42], and the 8-OH-dG residue amount was determined by HPLC-ED. 2′-Deoxyguanosine (dG) was measured in the same run of corresponding 8-OH-dG, and the results were expressed as the number of 8-OH-dG residues/10^6^dG residues. Data were shown as fold change (*n* = 3, ^∗^ *p* < 0.05, ^∗∗^ *p* < 0.01 by Student's test). (b) DNA was isolated from early and late passages of primary human keratinocyte cultures, and the 8-OH-dG residue amount was determined by HPLC-ED.2′-Deoxyguanosine (dG) was measured in the same run of corresponding 8-OH-dG, and the results were expressed as the number of 8-OH-dG residues/10^6^dG residues. Data were shown as fold change (*n* = 4, ^∗^ *p* < 0.05 by Student's test). In the inset, the 8-OH-dG amount was reported for each strain. (c, d) The best-fit line determined by linear regression was shown for each data series, with *R* ^2^ coefficient and Spearman correlation coefficient (*R*s). Two-tailed *p* value was reported in Result. (c) The number of cell doublings is expressed as %Life span (ratio of the number of cell doublings at selected passage on the total number of cell doublings) to take account of the life span variability among the 4 strains.

Accelerated senescence may be induced by many stress stimuli through p16 up-regulation. We previously have up-regulated or down-regulated p16 in primary human keratinocytes [42]. In those p16-overexpressing cultures, 8-OH-dG levels decreased compared to control (Figure 2(a)). On the contrary, p16 inactivation was able to induce 8-OH-dG increase (Figure 2(a)). Similar results were obtained in skin cells from Grossman and colleagues that underline a negative control of oxidative stress by p16. Thus, these results indicate that the observed 8-OH-dG accumulation in skin aging is not a causal effect of p16 increase.

The replicative senescence is a feature of tissues with high turnover, such as epidermis. In primary human keratinocyte cultures, replicative senescence is a gradual process controlled by a precise cell-doubling clock that counts cell divisions and is mediated by telomere shortening [34, 49–51]. Also telomeric DNA is susceptible to oxidative damage. Indeed, the triplet guanines in telomeric repeats contribute to increase the accumulation of oxidative lesions that are repaired with difficulty because of telomere configurations and, in turn, interfere with telomere length homeostasis [52, 53]. The end of the keratinocyte culture life span occurs when all stem cells have completed their clonal evolution, which is accompanied by p16 increase [34, 54].

Keratinocyte cultures obtained from 4 young donors have been serially cultivated and underwent 128.81, 140.47, 188.95, and 120.90 cell doublings, respectively. The steady-state level of 8-OH-dG was investigated at early (6.14, 9.19, 6.74, and 5.55 cell doublings, resp.) and late (86.17, 82.42, 101.41, and 86 cell doublings, resp.) passages, which correspond to 60–70% of culture life span. As shown in Figure 2(b), the mean steady-state level of 8-OH-dG was significantly higher at late passages (21.13-fold increase; *p* < 0.05). Specifically, 26.75-, 36-, 14.51-, and 7.25-fold increase of 8-OH-dG amount was observed in keratinocyte culture from late versus early passage of the same culture (Figure 2(b), inset). As shown in Figures 2(c) and 2(d), 8-OH-dG levels significantly correlated with the number of cell doublings in culture (*R*s = 0.7619, *p* = 0.0368) and with paraclone percentage (*R*s = 0.9341, *p* = 0.0022) by a linear relationship (*R* ^2^ = 0.7294, *p* = 0.0069; *R* ^2^ = 0.7013, *p* = 0.0086) and, therefore, with culture cell-doubling clock. Of note, during replicative senescence, the most intense p16 expression was evident later, around 70–80% of the culture life span [34, 42], suggesting that its increase may be a subsequent event of the oxidative damage accumulation. In keeping with our data, exposure of fibroblasts to 8-OH-dG base induces cell senescence by increased expression of p16 [55].

Thus, 8-OH-dG levels significantly increase during keratinocyte replicative senescence and clonal evolution. Altogether, these data suggest that the p16 increase and epidermal senescence may be a consequence of the impairment of the 8-OH-dG repair mechanisms.

### 3.3. DNA Repair System Efficiency in Aged and Senescent Primary Human Keratinocytes

The 8-OH-dG lesion is mainly removed from DNA by OGG1 through the coordinated action of several BER proteins in a multistep process. Thus, accumulation of 8-OH-dG might be considered as a diagnostic marker for BER malfunction [24]. BER may be subdivided into short-patch and long-patch subpathways depending on the length of the replaced oligonucleotide [25]. Whether 8-OH-dG lesions are present in resting DNA, the short-patch BER is the preferred repair system. When lesions persist or are formed during replication, the long-patch BER system is needed [25, 56, 57].

**Figure 3:**
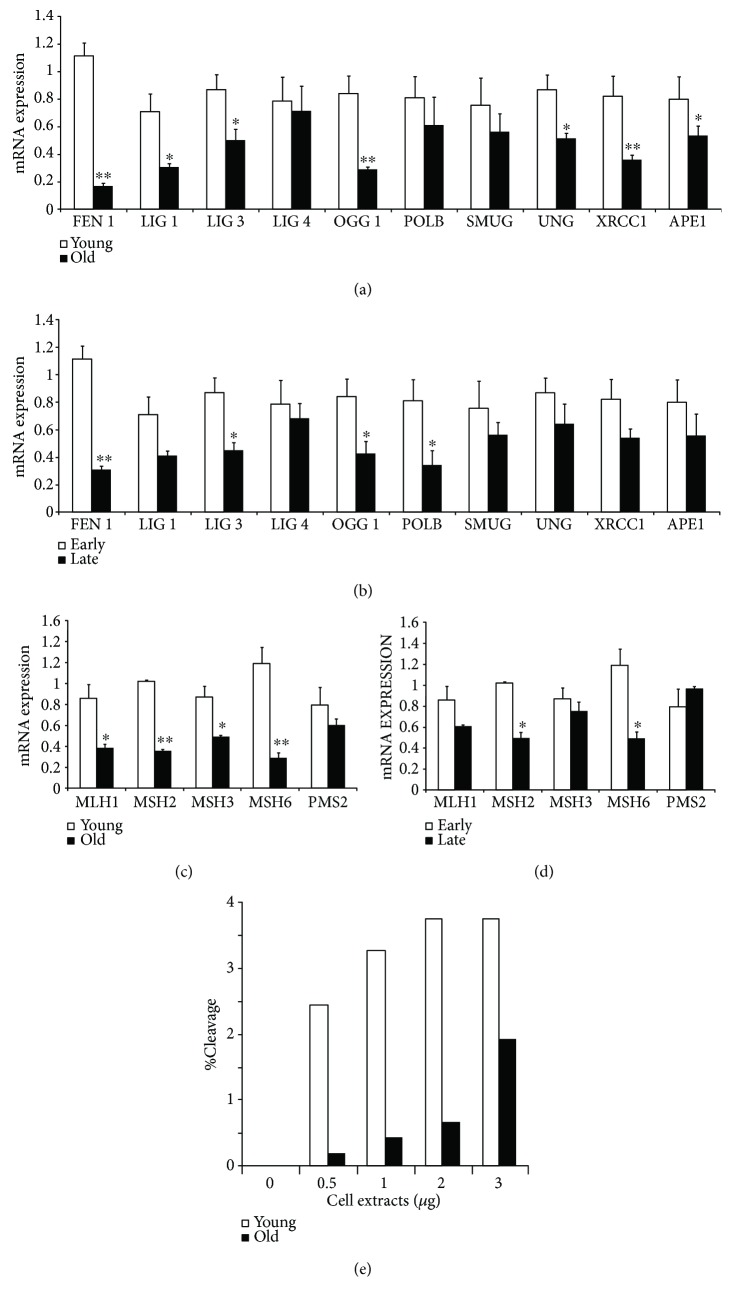
DNA repair system efficiency in 8-OH-dG removal in aged and senescent primary human keratinocytes. (a) BER enzyme expression was evaluated by quantitative RT-PCR analysis on RNA extracted from young and old donors at 2nd passage. Data were shown as fold change (*n* = 3, ^∗^ *p* < 0.05, ^∗∗^ *p* < 0.01 by Student's test). (b) BER enzyme expression was evaluated by quantitative RT-PCR analysis on RNA extracted from early and late passages of primary human keratinocyte cultures. Data were shown as fold change (*n* = 3, ^∗^ *p* < 0.05, ^∗∗^ *p* < 0.01 by Student's test). (c) MMR enzyme expression was evaluated by quantitative RT-PCR analysis on RNA extracted from young and old donors at 2nd passage. Data were shown as fold change (*n* = 3, ^∗^ *p* < 0.05, ^∗∗^ *p* < 0.01 by Student's test). (d) MMR enzyme expression was evaluated by quantitative RT-PCR analysis on RNA extracted from early and late passages of primary human keratinocyte cultures. Data were shown as fold change (*n* = 3, ^∗^ *p* < 0.05 by Student's test). (e) The ability of keratinocyte extracts from young and old donors (at 2nd passage) to repair the 8-OH-dG residues was tested in vitro using Rhodamine Green-labelled plasmid substrates containing 8-OH-dG. After incubation, plasmid DNA was submitted to SDS-PAGE to detect the fluorescence intensity of full-length DNAs and cleaved fragments by image analysis. The results were expressed as the cleavage percentage correlated to cell extract amount.

To investigate the mechanism responsible for the senescence-related 8-OH-dG accumulation, we examined the expression levels of BER components in primary human keratinocytes obtained from young and old donors. A global expression decrease of BER pathway factors has been found in cultures from old donors (Figure 3(a)). Particularly, a significant decrease was observed for most proteins involved in the short-patch process: OGG1 (*p* < 0.01), APE1 (*p* < 0.05), LIG3 (*p* < 0.05), and XRCC1 (*p* < 0.01). Moreover, the expression of UNG1 (*p* < 0.05) and some long-patch BER proteins, such as FEN1 (*p* < 0.01) and LIG1 (*p* < 0.05), were significantly decreased. Likewise, an expression decrease of BER enzymes has been found in late passages of the in vitro keratinocyte life span (Figure 3(b)). A significant decrease was observed for FEN1 (*p* < 0.01) and most proteins involved in the short-patch process, such as OGG1 (*p* < 0.05), POLB (*p* < 0.05), and LIG3 (*p* < 0.05).

As further defense strategy, the mismatch repair (MMR) system plays an important role in maintaining a low steady state of 8-OH-dG [58–60].

A significant expression decrease of MMR pathway factors has been found in cultures from old donors (Figure 3(c)): MLH1 (*p* < 0.05), MSH2 (*p* < 0.01), MSH3 (*p* < 0.05), and MSH6 (*p* < 0.01). Moreover, MSH2 and MSH6 were significantly down-regulated (*p* < 0.05) in late-passage keratinocytes (Figure 3(d)).

Altogether, these findings demonstrate that the expression of most DNA repair enzymes involved in 8-OH-dG removal significantly decreases during aging and replicative senescence of primary human keratinocytes.

To establish the differential ability of keratinocyte extracts from young and old donors to repair the 8-OH-dG residues, an in vitro repair test was carried out using Rhodamine Green-labelled plasmid substrates containing 8-OH-dG. A significant reduction of 8-OH-dG cleavage was observed in aged keratinocytes (Figure 3(e)).

Thus, 8-OH-dG accumulation can be ascribed to a global BER and MMR impairment in aged donors. This weakened efficiency of 8-OH-dG repair mechanisms is correlated to replicative cell senescence.

### 3.4. Expression of OGG1 Isoforms in Aged Primary Keratinocytes

The impairment of BER activity in aged keratinocytes can mostly be due to the down-regulation of OGG1 enzyme that catalyzes the first step of the repair process [25].

**Figure 4:**
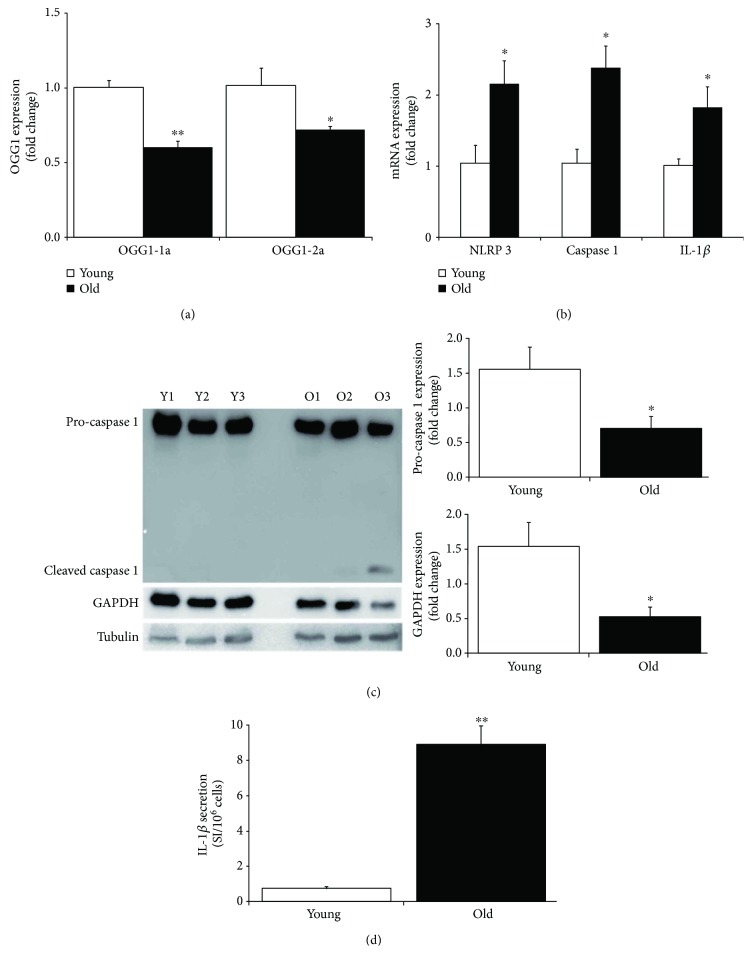
OGG1 isoform expression and inflammation in aged primary keratinocytes. (a) OGG1-1a and OGG1-2a expressions were evaluated by quantitative RT-PCR analysis on RNA extracted from young and old donors at 2nd passage. Data were shown as fold change (*n* = 3, ^∗^ *p* < 0.05, ^∗∗^ *p* < 0.01 by Student's test). (b) NLRP3, caspase 1, and IL-1*β* expressions were evaluated by quantitative RT-PCR analysis on RNA extracted from young and old donor cultures at 2nd passage. Data were shown as fold change (*n* = 3, ^∗^ *p* < 0.05 by Student's test). (c) Caspase 1 expression was evaluated by Western blot analysis on cell extract from young and old donor cultures at 2nd passage. Both pro-caspase 1 and cleaved caspase 1 forms were indicated. Expression levels of pro-caspase 1 and GAPDH proteins were evaluated by densitometric analysis and normalized by tubulin levels. Data were shown as fold change (*n* = 3, ^∗^ *p* < 0.05 by Student's test). (d) IL-1*β* secretion was assayed by antibody arrays using supernatants collected from young and old donor cultures at 2nd passage. Signal intensity (SI) was obtained by densitometric analysis and normalized with cell number (*n* = 3, ^∗∗^ *p* < 0.01 by Student's test).

The OGG1 gene contains eight exons that can be alternatively spliced at the C-terminal region to generate a number of different isoforms classified into two major groups, type 1 and type 2. Type 1 alternative splice variants end with exon 7 and type 2 end with exon 8. All variants share the N-terminal region in common, which contains a mitochondrial targeting signal essential for mitochondrial localization [61]. Only OGG1-1a isoform contains a nuclear localization signal at its C-terminus, which preferentially directs this isoform to the nucleus, whereas the other isoforms are localized in mitochondria [62]. The two major isoforms in human cells are OGG1-1a and OGG1-2a. Here, we investigated the expression of both isoforms in primary human keratinocytes. A significant expression decrease of OGG1-1a and OGG1-2a has been found in cultures from old donors (Figure 4(a)).

Thus, the impairment of BER initiation activity can be ascribed to down-regulation of both OGG1 isoforms.

### 3.5. Nucleotide-Binding Domain and Leucine-Rich Repeat Pyrin Domain Containing 3 (NLRP3) Inflammasome Activation in Aged Primary Keratinocytes

OGG1-2a prevents the activation of the nucleotide-binding domain and leucine-rich repeat pyrin domain containing 3 (NLRP3) inflammasome and IL-1*β* production [31]. Oxidized mitochondrial DNA is released into the cytoplasm where it binds to and activates the multiprotein complex NLRP3 [63] that, in turn, recruits and activates caspase 1, leading to cleavage and activation of IL-1*β* and IL-18 precursors [64, 65]. Keratinocytes are the major source of cytokines in the skin and play a central role in the inflammation and in adaptive immune responses [64]. Specifically, the inflammasome-dependent activation and secretion of IL-1*β* occur in keratinocytes following several stress and DNA damage [64, 66, 67].

Here, we observed a significant increase of NLRP3, pro-caspase 1, and pro-IL-1*β* mRNA steady-state levels in primary keratinocyte cultures from elderly donors (Figure 4(b)). Epidermis contains low levels of inactive precursor forms of IL-1*β*, which accumulate in the cell cytoplasm without active secretion. IL-1*β* requires caspase 1-mediated proteolysis for its maturation and secretion. Likewise, caspase 1 is activated by cleavage that releases a small active subunit [66]. Western blot displayed a significant decrease of pro-caspase 1 in all aged keratinocytes. Interestingly, the cleaved active caspase 1 form can be detected in unstimulated keratinocytes from two elderly donors (Figure 4(c)). Of note, the expression of GAPDH, which is a caspase 1 proteolytic target [68], was mainly reduced in those samples that display the mature form of caspase 1. This finding indicated that the cleaved caspase 1 characterizing aged keratinocytes was active and able to process its targets. Consistent with inflammasome activation [66, 67], primary keratinocyte cultures from elderly donors displayed a significant increased secretion of IL-1*β* compared to cultures from youngs in unstimulated condition (Figure 4(d)).

Altogether, these data demonstrate a constitutive activation of NLRP3 inflammasome in aged keratinocytes in parallel to the OGG1-2a down-regulation. These findings raise the possibility that the decrease of the oxidative DNA damage repair efficiency is able to induce the inflammasome activation during skin aging.

### 3.6. Posttranscriptional Modulation of OGG1-2a

The detected OGG1-2a transcript down-regulation in keratinocytes from elderly donors may be due to transcriptional and/or posttranscriptional modulation. Although transcriptional modulation of OGG1 activity has been investigated [27], the events involved in posttranscriptional modulation have not been analyzed in deep.

Oxidative stress induces the expression of several miRNAs that are critical for finely tuned posttranscriptional regulation of their target genes [29]. Among these miRNAs, miR-200 family members have been established as key regulators of epithelial phenotype and cellular senescence [69–71]. For miRNA-mRNA-mediated inhibition, a complete pairing between the 3′-untranslated regions (UTRs) of the RNA target and the seed sequence of the miRNA (i.e., a region centered on nucleotides 2–7) is required. The miR-200 family consists of five members that can be divided into two functional groups according to their seed sequences: miR-200b, miR-200c, and miR-429 belong to functional group I whereas miR-141 and miR-200a to functional group II [69]. The correlation between miR-200 family and OGG1 has not yet been investigated. However, OGG1 has been found target of miR-4673 [32] and miR-33a [31]. Of note, the miR-33a-mediated down-regulation of OGG1-2a in human and mouse cells resulted in increased 8-OH-dG accumulation, activation of NLRP3 inflammasome, and increased IL-1*β* production [31].

The seed pairing between miRNA and mRNA target is a necessary requirement for most target prediction algorithms. In silico analyses indicated that OGG1-2a isoform is a potential target of both miR-33a and miR-200a. Specifically, computational miRNA target analysis revealed that OGG1-2a has two predicted seed sequences for miR-33a and one seed sequence for miR-200a in its 3′-UTR (Supplemental Figure 1A).

**Figure 5:**
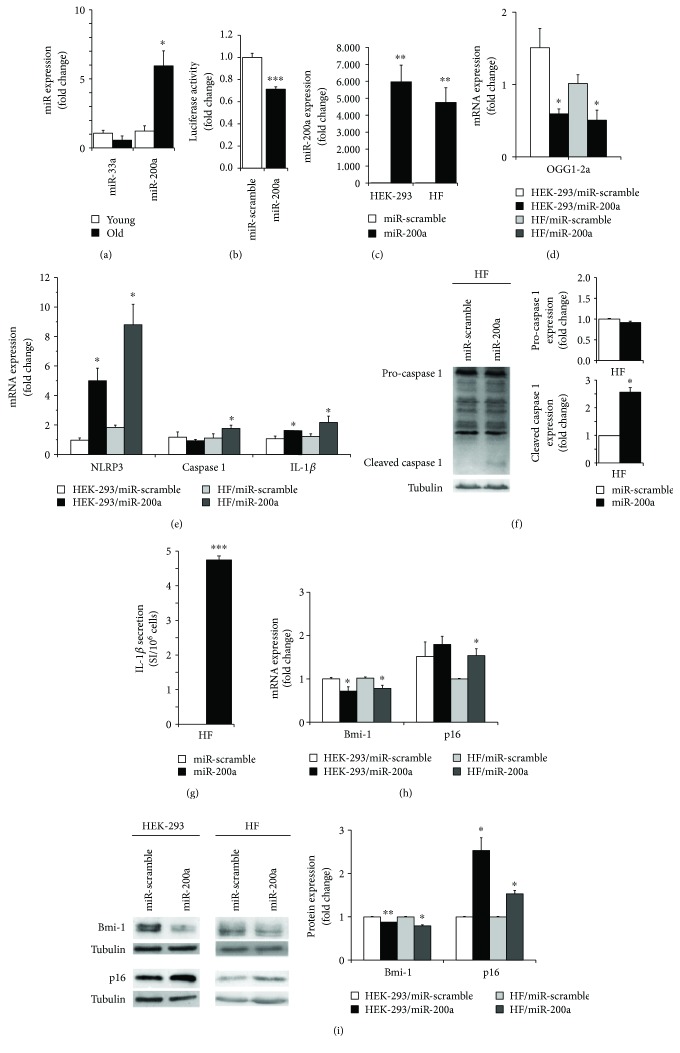
Posttranscriptional modulation of OGG1-2a. (a) miR-33a and miR-200a expressions were evaluated by quantitative RT-PCR analysis on RNA extracted from young and old donor cultures at 2nd passage. The miRNA levels were normalized using the U6 (*n* = 3, ^∗^ *p* < 0.05, by Student's test). (b) HEK 293 cells were cotransfected with firefly luciferase construct containing the 3′-UTR sequence of OGG1-2a gene and with a plasmid encoding either miR-200a or miR-scramble sequence. Values were normalized according to Renilla luciferase activity. Data were shown as fold change (*n* = 3 in triplicate, ^∗∗∗^ *p* < 0.0001, by Student's test). (c) miR-200a expression was evaluated by quantitative RT-PCR analysis on RNA extracted from miR-200a or miR-scramble transduced HEK 293 cells and primary human fibroblasts. Data were shown as fold change (*n* = 3, ^∗∗^ *p* < 0.01 by Student's test). (d) OGG1-2a, NLRP3, caspase 1, and IL-1*β* expressions were evaluated by quantitative RT-PCR analysis on RNA extracted from miR-200a or miR-scramble transduced HEK 293 cells and primary human fibroblasts. Data were shown as fold change (*n* = 3, ^∗^ *p* < 0.05 by Student's test). (e) Caspase 1 expression was evaluated by Western blot analysis on cell extract from miR-200a or miR-scramble transduced primary human fibroblasts. Both pro-caspase 1 and cleaved caspase 1 forms were indicated. Expression levels of pro-caspase 1 and cleaved caspase 1 proteins were evaluated by densitometric analysis and normalized by tubulin levels. Data were shown as fold change (*n* = 3, ^∗^ *p* < 0.05 by Student's test). (f) IL-1*β* secretion was assayed by antibody arrays using supernatants collected from miR-200a or miR-scramble transduced primary human fibroblasts. Signal intensity (SI) was obtained by densitometric analysis and normalized with cell number (*n* = 3, ^∗∗∗^ *p* < 0.001 by Student's test). (g) Bmi-1 and p16 expressions were evaluated by quantitative RT-PCR analysis on RNA extracted from miR-200a or miR-scramble transduced HEK 293 cells and primary human fibroblasts. Data were shown as fold change (*n* = 3, ^∗^ *p* < 0.05 by Student's test). (h) Bmi-1 and p16 expressions were evaluated by Western blot analysis on cell extract from miR-200a or miR-scramble transduced HEK 293 cells and primary human fibroblasts. Expression levels of Bmi-1 and p16 proteins were evaluated by densitometric analysis and normalized by tubulin levels. Data were shown as fold change (*n* = 3, ^∗^ *p* < 0.05, ^∗∗^ *p* < 0.01 by Student's test).

Unexpectedly, we found that miR-33a was down-regulated whereas miR-200a was strongly up-regulated in primary keratinocytes from elderly donors (Figure 5(a)), suggesting that the latter miRNA may be responsible of OGG1-2a down-regulation during skin aging.

To demonstrate that OGG1-2a is a direct target of miR-200a, HEK 293 cells have been cotransfected with a construct containing the OGG1-2a 3′-UTR downstream of a luciferase open reading frame and either with miR-200a or a miR-scramble. Relative luciferase activity was significantly down-regulated (~29%) upon miR-200a overexpression (Figure 5(b)).

Thus, OGG1-2a is a direct target of miR-200a that increases with primary keratinocyte aging.

### 3.7. miR-200a Modulates OGG1-2a, NRLP3, IL-1*β*, Bmi-1, and p16 Expression

To investigate whether miR-200a up-regulation was able to modulate the senescence and inflammation players characterizing aged keratinocytes, we took advantage of the easily transfectable HEK 293 cell line. Moreover, we used also primary human fibroblasts (HFs) since the inflammasome NLRP3 mediates the IL-1*β* secretion in this cell type following endogenous and exogenous stimuli and regulates important aspects of tissue repair and fibrosis [64]. Both HEK 293 cells and primary human fibroblasts have been transfected with miR-200a or a miR-scramble. Indeed, the inflammasome NLRP3 mediates the IL-1*β* secretion in fibroblasts.

As expected, following miR-200a expression (Figure 5(c)), a significant decrease of OGG1-2a expression was observed in both cell types (Figure 5(d)). Of note, miR-200a overexpression induced also a significant increase of NRLP3, caspase 1, and IL-1*β* expression (Figure 5(e)) mainly in fibroblasts, suggesting a role in the initiation of the inflammasome activation. Western blot analysis displayed a significant increased expression of cleaved form of caspase 1 (Figure 5(f)) and secretion of IL-1*β* (Figure 5(g)) following miR-200a overexpression in human fibroblasts, demonstrating the activation of NLRP3 inflammasome. Similar trend of expression was observed also in HEK 293 cells (Supplemental Figure 1B).

Bmi-1 is a well-known p16 repressor [72] with a fundamental role in human epidermal aging [35, 42]. Indeed, Bmi-1 is a member of the polycomb repressor complex 1 that mediates gene silencing by regulating chromatin structure and is critical for self-renewal of both normal and cancer stem cells [73]. Interestingly, Bmi-1 possesses a seed sequence for miR-200a, although the relation between miR-200a and Bmi-1 has not yet been analyzed.

Following miR-200a expression, Bmi-1 expression significantly decreased at both mRNA and protein levels (Figures 5(h) and 5(i)) in HEK 293 cells and primary human fibroblasts. Simultaneously, a significant increase of the senescence mediator p16 was observed (Figures 5(h) and 5(i)).

Altogether, these data demonstrate that miR-200a is able to down-regulate OGG1-2a and modulates the NLRP3/IL-1*β* and the Bmi-1/p16 axes. These findings indicate an active multiple role of miR-200a in aging establishment.

## 4. Discussion

Senescent cells are more commonly found in aged tissues and at sites of most age-related pathologies, both degenerative and hyperplastic [5, 47]. Cellular senescence is one of the main mechanisms that prevent genomic instability. However, senescent cells remain viable and persist for extended periods in the tissue contributing to stress responses after the onset of senescence. Chronic inflammation associated with cell senescence stimulates large production of ROS, causing, in turn, additional genetic instability. Thus, the proteins involved in DNA repair, cell cycle control, and inflammation interplay to maintain tissue homeostasis [27, 74, 75].

Oxidative stress and inflammation decrease the activity of OGG1 besides increasing the levels of 8-OH-dG [28, 76–79]. The OGG1 activity returned to normal levels once the redox cellular status is normalized [28]. OGG1 is a key enzyme of the BER system, and its dysfunction fosters DNA oxidative damage, mainly in mitochondrial DNA, leading to genomic instability [27]. Mitochondria are a major site of ROS production, and in turn, mitochondrial DNA is a major oxidative damage target because of its close proximity to the respiratory chains and the lack of protective histones [80]. The OGG1 gene generates different isoforms by alternative splicing. OGG1-1a and OGG1-2a isoforms are prevalent in human cells. OGG1-1a is mainly involved in nuclear BER activity [81]. Conversely, OGG1-2a has been demonstrated to be an important factor for oxidative BER repair of mitochondrial DNA in human cells [82], although OGG1-2a protein isolated from *E. coli* exhibits low or none oxidative DNA repair activity [83, 84]. Indeed, the enzymatic activities of the OGG1 proteins may be modulated by posttranslational modifications [27, 85]. Therefore, OGG1-2a proteins may be more active in whole human cells than following expression in *E*. *coli*. Moreover, OGG1-2a may also modulate mitochondrial DNA repair through a structural role via its interaction with the complex I factor NDUFB10 [82]. Down-regulation of mitochondrial OGG1 in accelerated senescence (SAM-P/8) mice may promote brain aging, through the accumulation of mitochondrial DNA damage [86]. Mitochondrial OGG1 deficiency or reduction leads to increased 8-OH-dG accumulation that induces NLRP3 activation and IL-1*β* secretion in mouse and human cells [31].

Although signaling by DNA strand breaks has been demonstrated critical for senescence and neoplastic escape of epithelial cells [12], the role of oxidative DNA repair system has not fully investigated in primary human keratinocyte senescence.

In this study, we show that

- primary human keratinocytes from elderly donors are characterized by a significant accumulation of the oxidative base lesion 8-OH-dG, an impairment of oxidative DNA repair and a decrease of OGG1-2a expression;
- OGG1-2a is a direct target of the ROS-induced miR-200a;
- the levels of miR-200a significantly increase in aged keratinocytes.

Altogether, our data indicate that ROS overproduction may induce both oxidative DNA lesions and miR-200a expression in aged skin. Notably, its up-regulation disrupts a defense mechanism by inhibiting OGG1-2a expression that decreases the BER efficiency and, in turn, increases 8-OH-dG accumulation (Supplemental Figure 2). In keeping with our data, miR-200a has been found up-regulated during differentiation of human keratinocytes [87].

Furthermore, the results of the present study indicate that primary keratinocytes from elderly donors display NRPL3 inflammasome activation and IL-1*β* secretion. Mitochondrial ROS and OGG1-2a deficiency are critical for the activation of the NLRP3 inflammasome [31, 63] that is implicated in the chronic low-grade inflammation characterizing metabolic, inflammatory, and age-related diseases [88–91]. Genetic variations in the NLRP family are associated with susceptibility to several cutaneous inflammatory disorders, such as atopic dermatitis [92–94]. Notably, atopic dermatitis patients display 8-OH-dG levels significantly higher than in controls. 8-OH-dG levels correlate with disease severity index [8]. Moreover, NLRP3 has been reported as an important player in the initiation and promotion of nonmelanoma skin cancers that are commonly characterized by 8-OH-dG lesions [95, 96]. UV radiations induce the increase of 8-OH-dG lesions and inflammatory responses through increased ROS production in human keratinocytes [97, 98]. Notably, topical treatment with OGG1 enzyme affects UV-induced skin carcinogenesis [99]. Furthermore, the UV-induced DNA damage triggers NLRP3 inflammasome activation and IL-1*β* release in keratinocyte cultures. In keeping with these data, NLRP3 is increased in UV-exposed human epidermis in vivo [64, 66, 67]. The active form of IL-1*β* increases in senescent cells and plays a critical role in the onset of the SASP. Indeed, caspase 1 inhibitors reduce the expression of the early SASP factors. Moreover, the inflammasome activation and IL-1*β* signaling reinforces paracrine senescence in several tissues [2, 100]. Our results obtained in HEK 293 cells and primary human fibroblasts show NLRP3 activation and IL-1*β* secretion following miR-200a overexpression (Supplemental Figure 2). These findings suggest that the age-dependent miR-200a increase in human keratinocytes may induce NLRP3 inflammasome activation through OGG1-2a down-regulation.

p16 is a key regulator of keratinocyte senescence and is up-regulated in epidermal cells from elderly donors [35, 42]. We show that p16 expression in primary human keratinocytes during aging is directly proportional to 8-OH-dG accumulation. Thus, the oxidative damage displays a linear relationship mainly with skin senescence instead of chronological individual aging. Altogether, these data point to 8-OH-dG as a biomarker of human skin aging. In keeping with [101], we observed a negative control of oxidative stress by p16 in human keratinocytes (Supplemental Figure 2). Indeed, p16 may act to suppress tumorigenesis through cell cycle arrest, which facilitates the DNA damage repair, and the control of ROS accumulation that induces oxidative DNA damage [101]. Notably, we observe that 8-OH-dG levels significantly increase during keratinocyte replicative senescence and clonal evolution before p16 increase. According to our data, exposure of fibroblasts to 8-OH-dG base leads to cell senescence by increasing p16 levels [55]. Thus, p16 increase and keratinocyte senescence may be a consequence of the impairment of the 8-OH-dG repair mechanisms.

Interestingly, we found that miR-200a overexpression down-modulates also its putative target Bmi-1 and up-regulates p16 (Supplemental Figure 2). In keeping with these results obtained in HEK 293 cells and primary human fibroblasts, Bmi-1 expression levels decrease in human keratinocytes from old donors and its down-regulation strongly correlates with early expression of p16 [35]. Moreover, Bmi-1 overexpression is able to modulate p16 levels, delays cell senescence, and restores clonal ability of keratinocytes from elderly donors [35]. Interestingly, Bmi-1 possesses a seed sequence for miR-200a, although the relation between miR-200a and Bmi-1 has not yet been analyzed. However, miR-141 and miR-200c, which belong to miR-200 family, have been demonstrated to directly target Bmi-1 [71, 102, 103]. miR-200c suppresses cell growth and metastasis through Bmi-1 down-regulation in cancer cells [102, 103]. miR-141, which possesses the same seed sequence of miR-200a, induces senescence in human fibroblasts via posttranscriptional regulation of Bmi-1 [71]. Moreover, Bmi-1 transcriptionally down-regulates miR-141 and miR-200c completing an autoregulatory loop necessary for maintaining tissue homeostasis [104]. Altogether, these findings suggest that the age-dependent modulation of Bmi-1 and p16 in keratinocytes may be due, at least in part, to age-related miR-200a up-regulation.

Altogether, these findings point miR-200a as key player in primary human keratinocyte aging since its overexpression reduces oxidative DNA repair activity and may induce cell cycle arrest via p16 up-regulation and fuels chronic inflammation via NLRP3 activation (Supplemental Figure 2).

Our findings have important implications for skin aging and nonmelanoma skin cancer. Indeed, the SASP is a major player of detrimental effects of senescence. Thus, eliminating senescent cells and/or attenuating the SASP are attractive antisenescence strategies in both age-related pathologies and cancer therapy [2, 5]. Down-modulation of miR-200a expression might be a useful tool to improve DNA repair system and reduce the chronic inflammation in aged skin.

## Figures and Tables

**Table 1:** 

mRNA	Forward	Reverse
OGG1-1a	5′CCCCAGACCAACAAGGAAC3′	5′AGGTCGGCACTGAACAGC3′
OGG1-2a	5′CTGTGGGGACCTTATGCTG3′	5′TCCTGGCAGAAGATAAGAGGAC3′
NLRP3	5′GAT CTTCGCTGCGATCAACAG3′	5′CGT GCATTATCTGAACCCCAC3′
Caspasi 1	5′TTTCCGCAAGGTTC GATTTTCA3′	5′GGCATCTGCGCTCTACCATC3′
IL-1*β*	5′GCCCTAAACAGATGAAGTGCT3′	5′ACCAGCATCTTCCTCAGCTT3′
Bmi-1	5′GGAGACCAGCAAGTATTGTCC3′	5′GACCATTCCTTCTCCAGGTAT3′
p16	5′CCAACGCACCGAATAGTTACG3′	5′GCGCTGCCCATCATCATG3′
GUSB	5′TGCAGGTGATGGAAGAAGTG3′	5′TTGCTCACAAAGGTCACAGG3′
GAPDH	5′AGCCACATCGCTCAGACAC3′	5′GCCCAATACGACCAAATCC3′

## Supplementary Materials

Supplementary Materials Supplemental Figure 1: miR-33a and miR-200a seed sequences on OGG1-2a gene. (A) The miR-33a and miR-200a seed sequences and their positions at the OGG1 3′-UTR were indicated in this schematic representation. (B) Caspase 1 expression was evaluated by Western blot analysis on cell extract from miR-200a or miR-scramble transduced HEK 293 cells. Both pro-caspase 1 and cleaved caspase 1 forms were indicated. Expression levels of pro-caspase 1 and cleaved caspase 1 proteins were evaluated by densitometric analysis and normalized by tubulin levels. Data were shown as fold change (*n* = 3, ^∗^ *p* < 0.05 by Student's test). IL-1*β* secretion was assayed by antibody arrays using supernatants collected from miR-200a or miR-scramble transduced HEK 293 cells. Signal intensity (SI) was obtained by densitometric analysis and normalized with cell number (*n* = 3, ^∗^ *p* < 0.05 by Student's test). Supplemental Figure 2: a diagram of the functional effects of miR-200a on the oxidative DNA repair activity, growth arrest, and chronic inflammation. Aged keratinocytes are characterized by a significant accumulation of intracellular ROS and oxidative base lesion 8-OH-dG, impairment of BER, increase of miR-200a and p16 levels, and NLRP3 inflammasome activation. ROS overproduction induces both oxidative DNA lesions, such as 8-OH-dG, and miR-200a expression. Here, we show that OGG1-2a, a BER enzyme critical for 8-OH-dG repair, is a direct target of miR-200a. Moreover, miR-200a overexpression down-regulates OGG1-2a and induces IL-1*β* secretion through NLRP3 pathway. IL-1*β* plays a critical role in the onset of the SASP that is implicated in the chronic low-grade inflammation and, in turn, ROS accumulation. OGG1-2a down-regulation and oxidative DNA damage are able to activate the NLRP3 inflammasome. Thus, the observed IL-1*β* secretion may be due to OGG1-2a down-regulation following miR-200a overexpression. Furthermore, we show that miR-200a overexpression down-regulates Bmi-1 and induces p16. p16 is able to negatively control the oxidative stress and increases during keratinocyte replicative senescence following 8-OH-dG accumulation, suggesting that p16 increase may be subsequent to oxidative damage repair impairment. The repression of p16 by Bmi-1 is well established in keratinocytes. Of note, Bmi-1 may be considered a putative target of miR-200a. Indeed, it displays a seed sequence for miR-200a and is a demonstrated target of miR-141, a miR-200 family member that posses the same seed sequence of miR-200a. Thus, the age-dependent modulation of Bmi-1 and p16 in keratinocytes may be due, at least in part, to age-related miR-200a up-regulation. In conclusion, miR-200a plays a pivotal role in primary human keratinocyte aging, since its overexpression reduces oxidative DNA repair activity and may induce growth arrest via p16 up-regulation and fuels chronic inflammation via NLRP3 activation. Gray lines indicate already demonstrated mechanisms, gray dotted lines indicate hypothesized mechanisms, and black lines indicate the original data derived from this article. Red text indicates up-regulated molecules whereas blue text indicates down-regulated molecules in primary human aged keratinocytes.
