# Supplementary material for: miR-200a Modulates the Expression of the DNA Repair Protein OGG1 Playing a Role in Aging of Primary Human Keratinocytes
Source: Oxid Med Cell Longev. 2018 Mar 25;2018:9147326. doi: 10.1155/2018/9147326 (PMC5889889; doi:10.1155/2018/9147326)
Supplement: Supplementary Materials — Supplemental Figure 1: miR-33a and miR-200a seed sequences on OGG1-2a gene. (A) The miR-33a and miR-200a seed sequences and their positions at the OGG1 3′-UTR were indicated in this schematic representation. (B) Caspase 1 expression was evaluated by Western blot analysis on cell extract from miR-200a or miR-scramble transduced HEK 293 cells. Both pro-caspase 1 and cleaved caspase 1 forms were indicated. Expression levels of pro-caspase 1 and cleaved caspase 1 proteins were evaluated by densitometric analysis and normalized by tubulin levels. Data were shown as fold change (n = 3, ∗ p < 0.05 by Student's test). IL-1β secretion was assayed by antibody arrays using supernatants collected from miR-200a or miR-scramble transduced HEK 293 cells. Signal intensity (SI) was obtained by densitometric analysis and normalized with cell number (n = 3, ∗ p < 0.05 by Student's test). Supplemental Figure 2: a diagram of the functional effects of miR-200a on the oxidative DNA repair activity, growth arrest, and chronic inflammation. Aged keratinocytes are characterized by a significant accumulation of intracellular ROS and oxidative base lesion 8-OH-dG, impairment of BER, increase of miR-200a and p16 levels, and NLRP3 inflammasome activation. ROS overproduction induces both oxidative DNA lesions, such as 8-OH-dG, and miR-200a expression. Here, we show that OGG1-2a, a BER enzyme critical for 8-OH-dG repair, is a direct target of miR-200a. Moreover, miR-200a overexpression down-regulates OGG1-2a and induces IL-1β secretion through NLRP3 pathway. IL-1β plays a critical role in the onset of the SASP that is implicated in the chronic low-grade inflammation and, in turn, ROS accumulation. OGG1-2a down-regulation and oxidative DNA damage are able to activate the NLRP3 inflammasome. Thus, the observed IL-1β secretion may be due to OGG1-2a down-regulation following miR-200a overexpression. Furthermore, we show that miR-200a overexpression down-regulates Bmi-1 and induces p16. p16 is able to [file 9147326.f1.pdf]

Supplemental Figure 1 – miR-33a and miR-200a seed sequences on OGG1-2a gene

A) The miR-33a and miR-200a seed sequences and their positions at the OGG1 3' UTR were indicated in this schematic representation.

B) Caspase 1 expression was evaluated by Western blot analysis on cell extract from miR-200a or miR-scramble transduced HEK293 cells. Both pro-caspase 1 and cleaved caspase 1 forms were indicated. Expression levels of pro-caspase 1 and cleaved caspase 1 proteins was evaluated by densitometric analysis and normalized by tubulin levels. Data were shown as fold change (n=3, *p<0.05 by Student's test). IL-1β secretion was assayed by antibody arrays using supernatants collected from miR-200a or miR-scramble transduced HEK293 cells. Signal intensity (S.I.) was obtained by densitometric analysis and normalized with cell number (n=3, *p<0.05 by Student's test).

Supplemental Figure 2 – A diagram of the functional effects of miR-200a on the oxidative DNA repair activity, growth arrest and chronic inflammation.

Aged keratinocytes are characterized by a significant accumulation of intracellular ROS and oxidative base lesion 8-OH-dG, impairment of BER, increase of miR-200a and p16 levels, and NLRP3 inflammasome activation.

ROS over-production induces both oxidative DNA lesions, such as 8-OH-dG, and miR-200a expression. Here, we show that OGG1-2a, a BER enzyme critical for 8-OH-dG repair, is a direct target of miR-200a. Moreover, miR-200a overexpression down-regulates OGG1-2a and induces IL-1β secretion through NLRP3 pathway. IL-1β plays a critical role in the onset of the SASP that is implicated in the chronic low-grade inflammation and, in turn, ROS accumulation. OGG1-2a down-regulation and oxidative DNA damage are able to activate the NLRP3 inflammasome. Thus, the observed IL-1β secretion may be due to OGG1-2a down-regulation following miR-200a overexpression. Furthermore, we show that miR-200a over-expression down-regulates Bmi-1 and induces p16. p16 is able to negatively control the oxidative stress and increases during keratinocyte replicative senescence following 8-OH-dG accumulation, suggesting that p16 increase may be subsequent to oxidative damage repair impairment. The repression of p16 by Bmi-1 is well-established in keratinocytes. Of note, Bmi-1 may be considered a putative target of miR-200a. Indeed, it displays a seed sequence for miR-200a and is a demonstrated target of miR-141, a miR-200 family member that posses the same seed sequence of miR-200a. Thus, the age-dependent modulation of Bmi-1 and p16 in keratinocytes may be due, at least in part, to age-related miR-200a up-regulation.

In conclusion, miR-200a plays a pivotal role in primary human keratinocyte aging, since its over-expression reduce oxidative DNA repair activity, and may induce growth arrest via p16 up-regulation and fuels chronic inflammation via NLRP3 activation

Gray lines indicate already demonstrated mechanisms, gray dotted line indicate hypothesized mechanisms and black lines indicate the original data derived from this article. Red text indicates up-regulated molecules whereas blue text indicates down-regulated molecules in primary human aged keratinocytes.

SUPPLEMENTAL FIGURE 1

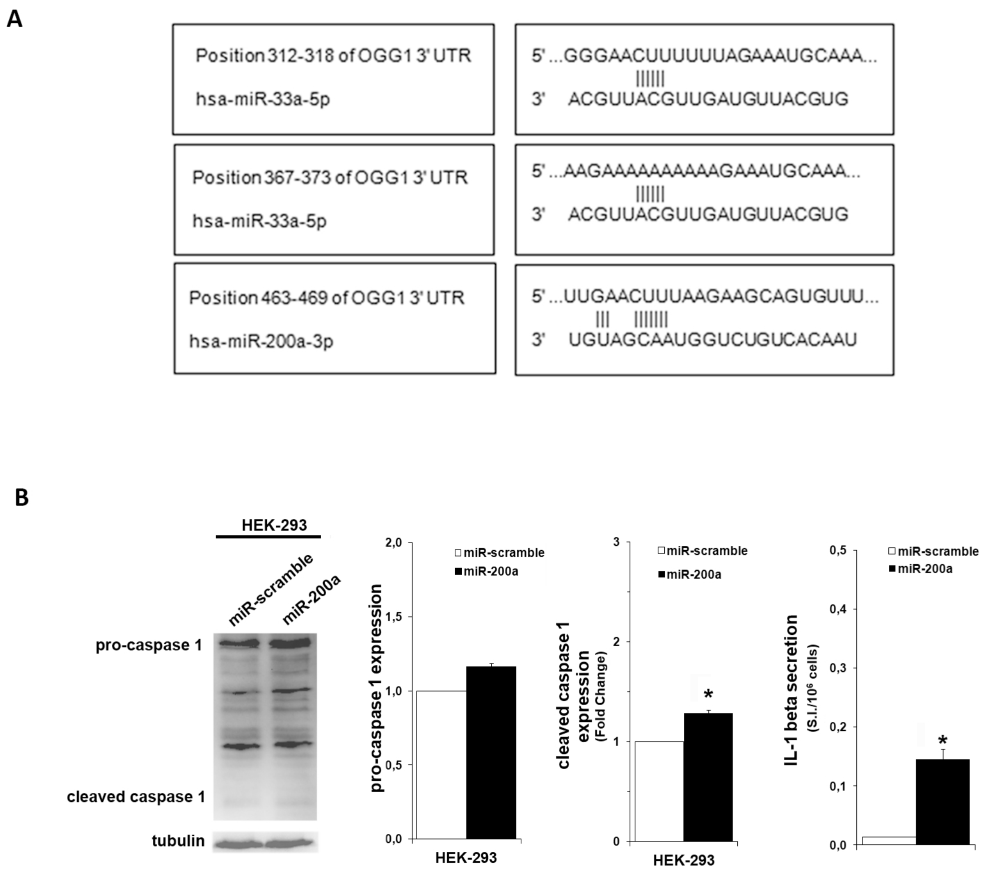

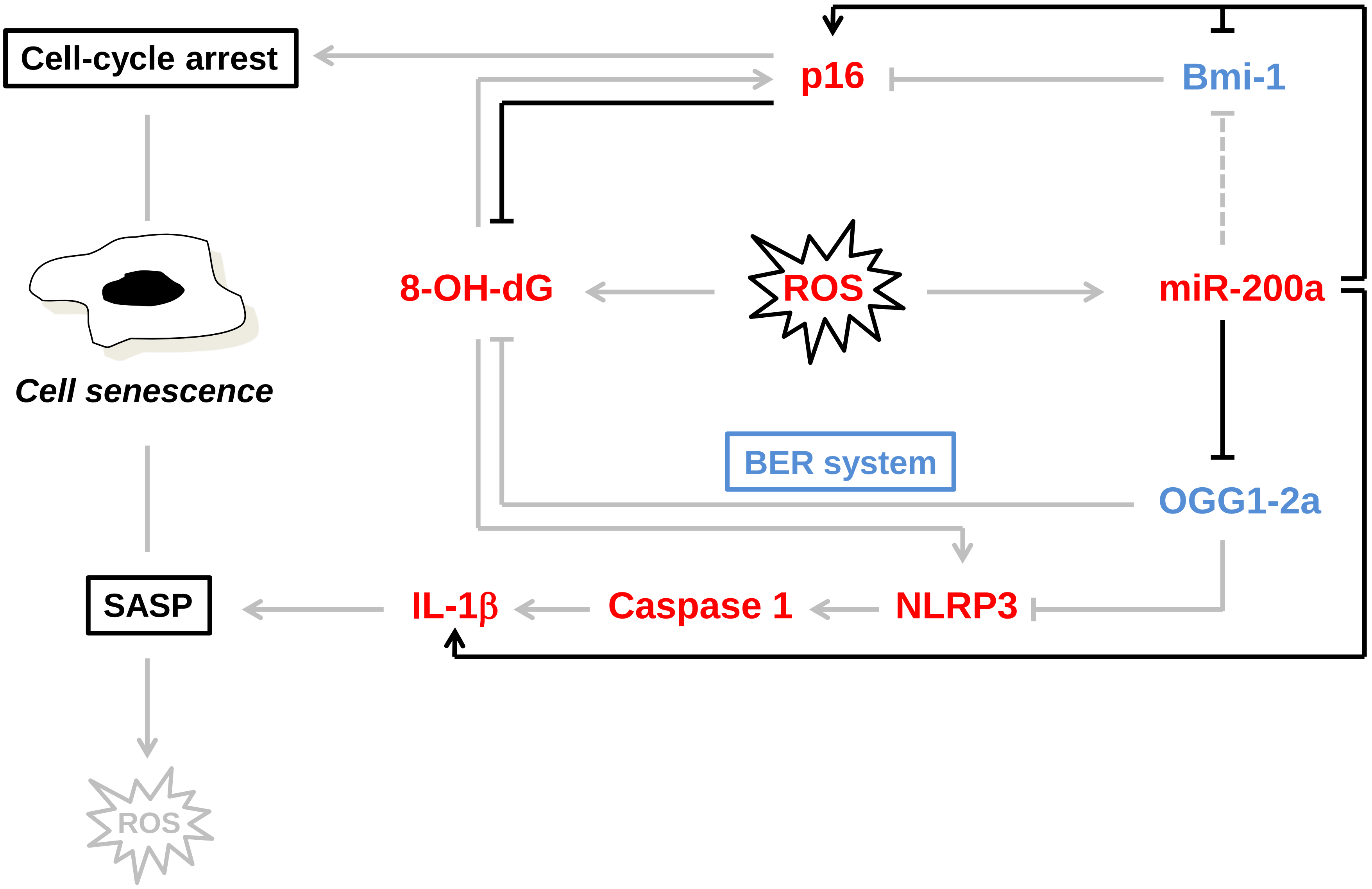
Cell-cycle arrest
p16
Bmi-1
8-OH-dG
ROS
miR-200a
Cell senescence
BER system
OGG1-2a
SASP
IL-1β
Caspase 1
NLRP3
ROS
